# Impact of Operating Time on Selected Tribological Properties of the Friction Material in the Brake Pads of Passenger Cars

**DOI:** 10.3390/ma14040884

**Published:** 2021-02-12

**Authors:** Andrzej Borawski

**Affiliations:** Faculty of Mechanical Engineering, Bialystok University of Technology, Wiejska 45C Street, 15-351 Białystok, Poland; a.borawski@pb.edu.pl

**Keywords:** mechanical engineering, coefficient of friction, abrasive wear factor, automobile brakes, Taguchi method

## Abstract

Braking systems have a direct impact on the safety of road users. That is why it is crucial that the performance of brakes be dependable and faultless. Unfortunately, the operating conditions of brakes during their operating time are affected by many variables, which results in changes in their tribological properties. This article presents an attempt to develop a methodology for studying how the operating time affects the value of the coefficient of friction and the abrasive wear factor. The Taguchi method of process optimization was used to plan the experiment, which was based on tests using the ball-cratering method. The results clearly show that the degree of wear affects the properties of the friction material used in the production process of brakes.

## 1. Introduction

The braking system is one of the most important elements of any vehicle. The health and life of the driver, passengers, and other road users may depend on the regularity and effectiveness of brakes. For this reason, numerous studies have been and will be conducted in order to identify problems related to the construction and operation of brakes, as well as improving the braking system [1,2,3].

The most common brakes in today’s vehicles are friction disc brakes, which replaced the older drum brake design [4,5]. Disc brakes use friction to convert mechanical, kinetic energy into heat. Thermal energy is then transferred to the atmosphere and to other parts of the braking system and the vehicle’s suspension [6,7].

Brake heating is an important and complicated process. The results of laboratory and simulation tests show that brake discs and pads are the elements that are subjected to the biggest amounts of heat [8,9,10,11]. The heat generated during braking, its speed of transfer, as well as the value of the coefficient of friction (COF), strictly depend on the type of materials used in the production of the braking system’s components [12,13,14]. The effectiveness of the brakes depends primarily on the value of the coefficient of friction between the pads and the disc. Unfortunately, the COF is not constant [15,16]. Large temperature changes of the operating components of breaks mean that the value of the COF between the disc and the pad, in some cases, drops to almost zero [17]. The humidity level near the abrasive pair is also significant. High humidity can lead to condensation and the formation of a water film. At low speeds of cooperation between the pad and the disc, the modulation of the coefficient of friction value can be as high as 30% [18].

The phenomenon of friction is closely related to wear. Today’s emphasis on environmental protection has made wear a popular research topic, especially in the case of friction materials used in the automotive industry. The reason is that wear products are released to the environment, causing pollution. Thus, the wear rate coefficient, the chemical composition, and the size of the particles emitted due to friction are significant. Such research was performed by Gawande et al. [19]. Additionally, the weight loss of asbestos-containing and non-asbestos pads was compared. The nature of the emitted particles was investigated by Sinhra et al. [20], whose study provided valuable information about the harmful effect of such emissions on living organisms. Moreover, it has been suggested that wear products are one of the reasons for the occurrence of airborne PM (particulate matters), which has been confirmed by other researchers [21,22]. The impact of wear products on human health was also investigated by Sarnat et al. [23] and Arden Pope et al. [24]. These authors found that such particles have a huge impact on the respiratory system and contribute significantly to serious disorders. In some cases, wear tests have a different background. Ramadan et al., for example, investigated the frictional effects of various samples in order to find a substitute for asbestos [25].

The presented analysis of literature shows that the results of research published so far focus primarily on determining the features and effects of the friction process, e.g., heating temperature, wear rate, the resulting surface geometry, or products of the friction process itself that are released to the environment, and their impact on living organisms. In these studies, “brand-new” samples were used (mostly prototypes, whose composition has not been widely used so far), and they had no contact with the actual operating conditions of brakes. This is important because difficult or even extreme working conditions resulting from frequent and significant temperature changes, as well as a corrosive environment (salt and water, especially in the winter) can permanently change the structure of the friction material, and, in consequence, the tribological properties of brake pads and discs. This results in a decrease in braking force, as well as larger distances required to stop the vehicle [26]. This article is an attempt to determine the influence of such conditions and operating time on the properties of the friction material, which has not been tried so far. The experiments described below assessed whether the tribological properties of brake friction elements change over time, and to what extent. In the future, there is a plan to test the wear products of particular samples in terms of size, shape, and chemical composition, but this is not the subject of this study.

An innovative research approach was applied, consisting in the use of the properly prepared ball-cratering method to determine the values of the coefficient of friction and the abrasive wear factor of association; the brake pad composite material–counter-sample. So far, research has been based mainly on the pin-on-disc method. In addition, pads exposed to normal exploitation were used for the production of samples; thus, the influence of natural conditions on the properties of the material was investigated.

## 2. Materials and Methods

**Figure 1:**
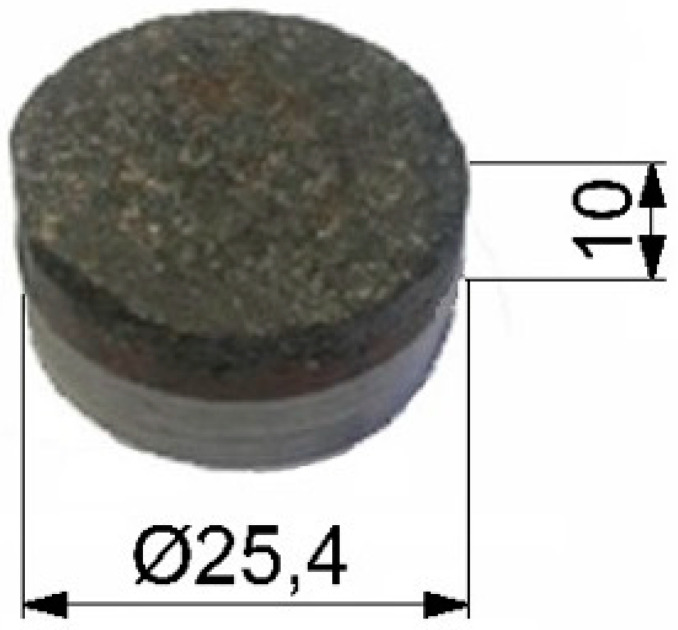
One of the samples used in research (mm).

The research objects were samples in the shape of a cylinder, 1” in diameter and a 10 mm thickness (Figure 1). Three groups of genuine brake pads, all produced by the same company for three different car models, were used to complete the samples. The first group consisted of brake pads for a small city car. The second group included pads dedicated to medium-class cars, while in the third, there were pads for a large, off-road vehicle. In each group, there were different types of pads—both brand-new and used ones in varying degrees of wear (from about 15% of wear to almost no lining at all) obtained by courtesy of an Authorized Service Station. In total, over 100 pads were used for testing.

**Figure 2:**
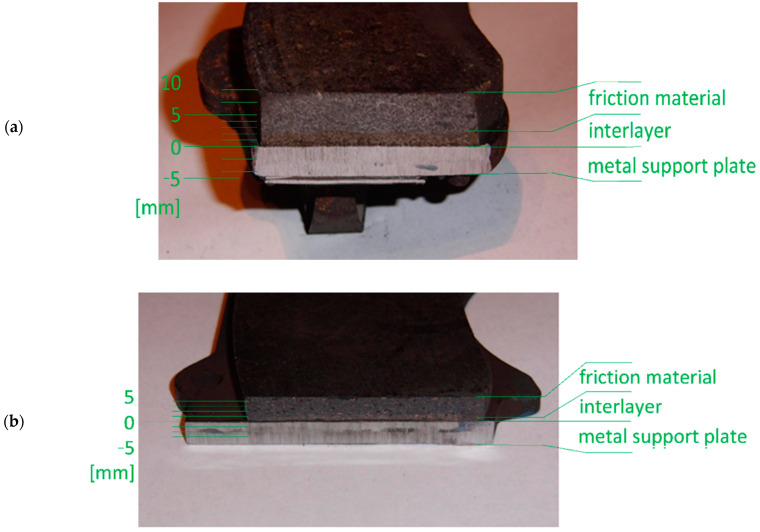
Cross-sections of brake pads used in the tests: (**a**) first group and (**b**) third group.

In the first group of pads, there are three layers that were important from the point of view of the performed tests, and all could be clearly distinguished (Figure 2a): the metal support plate, about a 3 mm thick binder layer (interlayer), and the friction material. In the second and third groups, the interlayer was much thinner (less than 1 mm, an example is shown in Figure 2b), and irregular. Therefore, in their case, the results presented below relate mainly to the support plate and friction material. The chemical composition of individual groups of pads and their layers was determined using the Phenom XL electron microscope (Thermo Scientific, Taren Point, Australia) equipped with an EDS (Energy Dispersive Spectroscopy) detector (SDD (Silicon Drift Detector) type). The collected data are presented in Table 1.

During the process of cutting the samples, it was important not to disrupt the friction lining structure, as this could affect the material properties and thus the test results. Therefore, techniques that did not generate high mechanical or thermal loads were used. The shape was pre-cut with a saw, then the desired shape was adjusted with a file. The sample was mounted throughout the entire treatment period between two 1” diameter rollers. For technological reasons resulting from the research methodology, the friction surface of the pad was polished manually. This allowed the surface unevenness resulting from the cooperation with the brake disc to be excluded.

**Figure 3:**
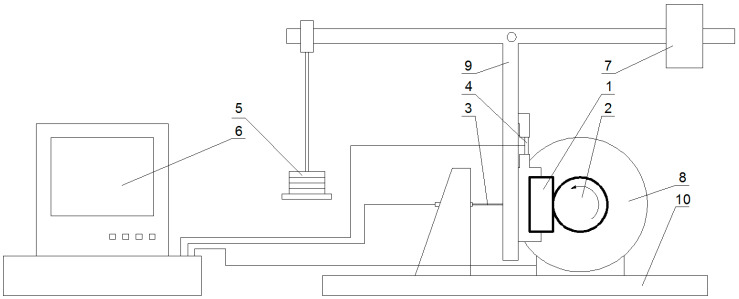
Diagram of the T-20 test stand: 1—sample, 2—counter-sample (sphere), 3—displacement sensor, 4—strain gauge for measuring friction force, 5—load, 6—computer, 7—counterweight, 8—electric motor, 9—swivel leaver, 10—deck.

Among the many research methods available in the field of tribology [27,28,29], a method called ball-cratering was selected in this experiment. This method is often used to study the tribological properties of materials. The association of the friction pair, which, in this case, is a flat-surface sample and a ball-shaped counter-sample, is not an ideal reflection of the actual operating conditions of the brakes. However, due to the quasistatic nature of the planned tests (i.e., with the input parameters unchanged during the tests), the decision was made to use the advantages of the ball-cratering method (which include, e.g., the ease of sample preparation, a good repeatability of the results, or a short time per single test) and to try using it to determine the values concerned. The T-20 test station (Instytut Technologii Eksploatacji, Radom, Poland) was used for this purpose (Figure 3).

In this device, the tested sample (1) is mounted in a holder located on the vertical arm of the lever, and its mass is balanced by a counterweight (7) located at one end of the horizontal lever arm. On the other end, a pan is hung, on which a load (5) is placed, ensuring that the pressure of the sample against the counter-sample (the counter-sample used in the tests was a ball with a diameter of 1 “(25.4 mm) made of 17HNM (EN: 18CrNiMo7-6) steel (2). The lengths of the arms of the lever are the same, so the clamping force is identical to the gravity force resulting from the pressure of the load set on the pan. The counter-sample is mounted on the shaft of an electric motor (8) (with adjustable speed). The ball rotates against the sample, forming a crater from the resulting friction. The crater is measured in order to determine the abrasive wear factor (*K_c_*). The strain gauge (4) located above the sample holder allows the direct measurement of the friction force during tests.

It should be noted that some researchers recommend using, e.g., rubber balls and loading them on both sides with the tested samples [30] or immersing the friction node in an abrasive suspension [31]. Analyzing the real-life working conditions of brakes in a vehicle, which rub in “dry” conditions, it was decided to perform the tests in the traditional way.

Many scientists carry out their research according to a comprehensive plan, which is time-consuming [32,33,34]. By using the selection plan, the time of research could be significantly shortened, without endangering the quality of the results [34,35,36]. During the planning process, the correct selection of input parameters is crucial [37,38]. In the case under consideration, these parameters are the rotational speed of the ball, the load, and the distance (length) of friction [39]. Analyzing various planning methods, it was decided to use the Taguchi method of process optimization, which is commonly used in this type of research [40,41]. It is also used in other branches of science, e.g., for planning construction experiments [42], the optimization of burnishing processes [43], or in biological sciences [44].

The first step of planning, according to the Taguchi method, was to perform several preliminary tests. One sample from each of the three groups of pads was selected for their implementation. The initial input parameters of the tests were randomly set, and the obtained results allowed for an initial estimate of the ranges of input parameters in which satisfactory crater sizes were obtained. They also allowed the development of an orthogonal array (Table 2).

**Figure 4:**
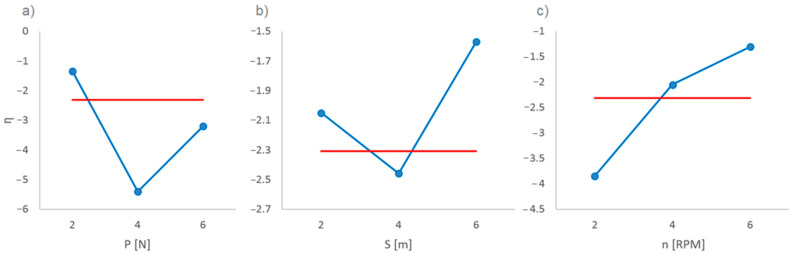
Dependence of the *η* parameter on: (**a**) load *P*, (**b**) friction distance *S*, and (**c**) counter-rotational speed *n*.

There were three variable input parameters, and each of them could have three values, so the preliminary studies were divided into nine groups. Each of the nine preliminary tests was performed five times. Next, following the criterion “the smaller, the better”, and using the results obtained in preliminary tests, the *η*(*y*_i_) parameter was calculated, which determines the ratio of the signal (in this case the friction of the ball and the sample) to the interference:

(1) $$\eta (y_{i})=-10{\mathrm{lg}}_{10}\left(\frac{1}{m}\sum_{i=1}^{m}y_{i}^{2}\right)$$

where: *m*—number of measurements (in this case *m* = 5) and *y_i_*—result of preliminary tests. The calculation results allow for the determination of the optimal (giving repeatable, low-error results) input parameters of the proper experiment for all three groups of pads (Figure 4). These are the final values: *P* = 2N, *S* = 150 m, and *n* = 150 RPM (revolutions per minute). Considering the diameter of the ball (1”), it is easy to calculate the linear velocity at the contact point, which is *v_l_* = 0.0319 m/s. Moreover, it should be noted that the tests were carried out in an indoor laboratory with a constant temperature of about 21 °C and an approximately 45% air humidity. For greater clarity, the parameters of the experiment are summarized in Table 3.

## 3. Results and Discussion

**Figure 5:**
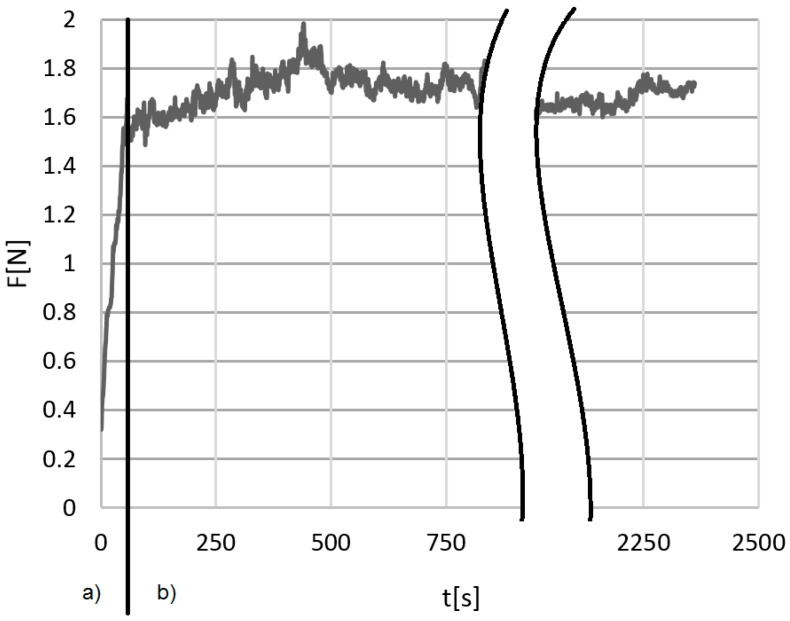
Exemplary time profile of the friction force *F* obtained during one of the tests: (**a**) running-in and (**b**) proper measurement period.

As a result of the study, numerical data from the direct measurement of the friction force were obtained. The T-20 stand software automatically saved the value every 0.5 s, which gave nearly 2500 measurement points and allowed the determination of the friction force time profiles (Figure 5). Their course makes it possible to distinguish the run-in period and the period of specific (correct) friction process.

**Figure 6:**
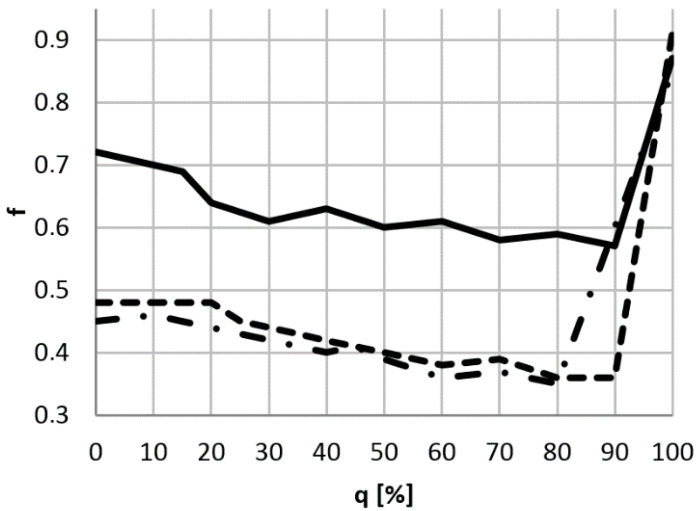
Dependence of the coefficient of friction *f* of brake pads from the degree of wear *q*: — first group; - - second group; - • - third group.

For each of the tests, using the value of force *F* measured in the period of specific friction process (ignoring data from the running-in period), the arithmetic average was calculated. The coefficient of friction *f* was determined by dividing the previously obtained value by the load *P*, (Figure 6). The collected data also allowed the calculation of the standard deviation (Table 4).

The test results of the first group of samples showed that the average COF of the friction material is high, and its value is about 0.64. For the interlayer (binder), the coefficient oscillates around 0.58. The second type of pads had a much thinner interlayer with a coefficient of friction similar to the friction material, on average around 0.42. The third type of pads also had a thin interlayer, but it had a much higher average COF of about 0.60. In this group, the friction material had an average coefficient of friction of approximately 0.4. All groups of pads have two common features: the largest friction coefficient (0.85–0.91) was found in completely worn pads (when friction results from the metal support plate), and a decrease in the friction coefficient over time. The largest decrease, by close to 0.15, was observed in the first group. In the second and third groups, the decrease in the coefficient of friction was about 0.1.

**Figure 7:**
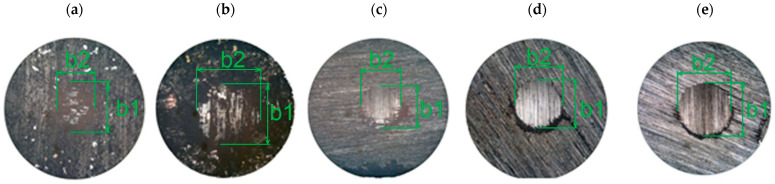
Sample photos of craters created during research: (**a**) first group of samples, *q* = 40%; (**b**) second group of samples, *q* = 15%; (**c**) second group of samples, *q* = 70%; (**d**) third group of samples, *q*= 80%; (**e**) third group of samples, *q* = 100%.

As was stated previously, the results of this study include craters formed in samples (examples of craters are shown in Figure 7). Measurement of their size in two planes (*b1*—in the direction of friction and *b2*—perpendicular to the direction of friction) allows for the arithmetic mean of the crater size to be obtained—*b*.

**Figure 8:**
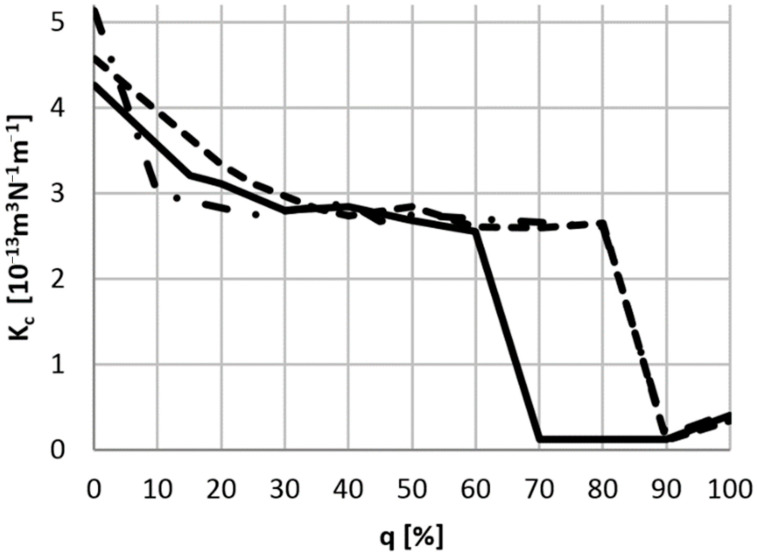
Dependence of the abrasive wear factor *K_c_* of brake pads from the degree of wear *q*: − first group; − − second group; − • − third group.

For dry friction, the values of the abrasive wear factor *K_c_* are commonly calculated according to the Archard formula [45,46,47]:

(2) $$K_{c}=\pi \frac{b^{4}}{64R\times S\times P}$$

where: *b*—arithmetic average of the measurements of the crater diameter in the direction of the sphere rotation and in the perpendicular direction (for this purpose the OLYMPUS BX51M microscope (Olympus IMS, Waltham, MA, USA) and Brinell magnifying glass JC-10 (Physical Test Solutions, Culver City, CA, USA) were used); *R*—the counter-sample radius. The results of the calculations of *K_c_* and the standard deviation for all three groups of pads are shown in Figure 8 and Table 5.

Test results show that all three groups of tested pads have similar characteristics in terms of wear intensity. All brand-new pads were characterized by the highest *K_c_* value, which was caused by the running-in process, during which the most intense wear occurs. After reaching the proper friction, the value of the coefficient stabilizes to approximately 2.9·10^−13^·m^3^·N^−1^·m^−1^. There was also a significant drop in the abrasive wear factor value for pads with a completely worn friction layer and also when the contact surface was the interlayer (for the first group, it was about 60–70% and for the second and third group about 90%). A slight increase in *K_c_* was noticed for completely worn pads.

The obtained results are characterized by a similar shape and angle of inclination, especially in the friction range of the first and second layer. This leads to a simple mathematical relationship in this respect:

- for the friction coefficient:

(3) $$f_{q}=-15,66q\times 10^{-6}+f_{0}$$

where: *f_q_*—a coefficient of friction of a brake pad worn to *q* and *f*_0_—a coefficient of friction of a brand-new brake pad,

- for the abrasive wear factor:

(4) $$K_{cq}=6q^{2}\times 10^{-4}-6,87q\times 10^{-2}+K_{c0}$$

where: *K_cq_*—abrasive wear factor of a brake pad worn to *q* and *f*_0_—abrasive wear factor of a brand-new brake pad.

Thanks to these interrelations, it is possible to predict the changes of the COF and abrasive wear rate values of the brake pads at various stages of wear based on only one measurement—a brand-new pad. Of course, the result will be correct only during the friction period with the first and second layer. In the presented case, it will be for about 70–90% of the total lifetime of the pad.

## 4. Conclusions

This study proposes a methodology for testing the coefficient of friction and the abrasive wear factor of the working elements of braking systems. Based on this methodology, almost four hundred samples taken from over one hundred brake pads were tested. For this purpose, three different models of genuine parts were used (brand-new and worn to varying degrees) from three different car models.

The test results allowed the estimation of the impact of operating time on the value of the coefficient of friction and the abrasive wear factor. It was found that:

1. the coefficient of friction for all tested brake pads decreases with the operation time;
2. two factors have the greatest impact on changes in the coefficient of friction: (1) atmospheric conditions and large changes in operating temperature causing a change in the structure of the pads’ layers (with the increase of wear, different tribological parameters were recorded in the same layer) and (2) a change in the composition of the material from which the pad is made of (depending on the degree of wear, the working surface is friction material, binder, or support plate);
3. the fastest wearout of the pads takes place when they are brand-new, which results from the “run-in” process;
4. the slowest wear of the pads was noticed when the friction surface was the interlayer.

From a safety point of view, information on the coefficient of friction is extremely important because it has a direct effect on the value of the braking force. However, the value of the *K_c_* factor says much about the lifetime of the pads. Information on the nature of changes in both parameters is valuable for vehicle manufacturers and users.

## Figures and Tables

**Table 1:** Composition of individual samples and their layers.

Brake Pads Group No.	Layer	Composition (% of Total Mass)
1	Friction material	Phenolic resin—21.31%, Steel fibers—4.16%, Glass fiber—7.98%, Cast iron fibers—4.73%, Silicon carbide—1.18%, Zeolits—5.84%, Zinc oxide—1.19%, Graphite—1.57%, Copper—5.96%, Barite—15.66%, Silicates—8.38%, Magnesium oxides—15.25%, Rubber particles—6.79%
	Binder layer (interlayer)	Phenolic resin—38.43%, Steel fibers—2.64%, Cast iron fibers—1.58%, Silicon carbide—0.49%, Zeolits—5.19%, Zinc oxide—0.82%, Graphite—1.57%, Barite—21.63%, Silicates—11.3%, Magnesium oxides—16.35%
	Support plate (backplate)	C—0.18%, Mn—1.38%, Si—0.2%, P—0.03%, S—0.02%, Fe—98.19%
2	Friction material	Phenolic resin—27.35%, Steel fibers—3.11%, Glass fiber—5.71%, Cast iron fibers—2.96%, Silicon carbide—0.82%, Zinc oxide—2.14%, Graphite—3.27%, Copper—8.19%, Barite—18.69%, Silicates—10.53%, Magnesium oxides—17.23%
	Binder layer (interlayer)	Phenolic resin—45.74%, Steel fibers—1.49%, Cast iron fibers—1.31%, Silicon carbide—0.39%, Zeolits—3.51%, Zinc oxide—1.69%, Copper—7.94%, Graphite—2.19%, Barite—14.93%, Silicates—4.83%, Magnesium oxides—15.98%
	Support plate (backplate)	C—0.17%, Mn—1.44%, Si—0.11%, P—0.03%, S—0.03%, Fe—98.22%
3	Friction material	Phenolic resin—27.83%, Steel fibers—2.97%, Glass fiber—5.52%, Cast iron fibers—2.19%, Silicon carbide—0.79%, Zinc oxide—2.44%, Graphite—3.73%, Copper—8.85%, Barite—18.71%, Silicates—11.02%, Magnesium oxides—15.95%
	Binder layer (interlayer)	Phenolic resin—47.27%, Steel fibers—1.41%, Cast iron fibers—1.22%, Silicon carbide—0.43%, Zeolits—3.54%, Zinc oxide—1.75%, Copper—8.22%, Graphite—2.11%, Barite—15.62%, Silicates—5.09%, Magnesium oxides—13.34%
	Support plate (backplate)	C—0.18%, Mn—1.42%, Si—0.19%, P—0.03%, S—0.02%, Fe—98.16%

**Table 2:** Orthogonal table of preliminary tests.

Preliminary Test No.	Load *P* (N)	Distance *S* (m)	Rotation Speed of Counter-Sample *n* (RPM)
1	2	50	38
2	2	100	80
3	2	150	150
4	4	50	80
5	4	100	150
6	4	150	38
7	6	50	50
8	6	100	150
9	6	150	80

**Table 3:** Experiment parameters.

Parameter	Value	Unit
Load	5	N
Distance	150	m
Rotation speed of counter-sample	150	RPM
Ambient temperature	21	°C
Humidity	45	%

**Table 4:** Test results of the coefficient of friction.

1st Group of Samples				2nd Group of Samples				3rd Group of Samples			
*q* (%)	Number of Tests Performed	Average *f* Value	Standard Deviation	*q (*%)	Number of Tests Performed	Average *f* Value	Standard Deviation	*q* (%)	Number of Tests Performed	Average *f* Value	Standard Deviation
0	12	0.72	± 0.051	0	12	0.48	± 0.018	0	12	0.45	± 0.045
~15	6	0.69	± 0.032	~20	8	0.48	± 0.021	~10	6	0.46	± 0.022
~20	18	0.64	± 0.048	~25	8	0.45	± 0.014	~25	14	0.43	± 0.031
~30	12	0.61	± 0.046	~35	6	0.43	± 0.033	~40	10	0.4	± 0.014
~40	15	0.63	± 0.038	~40	10	0.42	± 0.052	~45	8	0.41	± 0.016
~50	15	0.6	± 0.035	~50	12	0.4	± 0.015	~50	16	0.39	± 0.031
~60	18	0.61	± 0.047	~60	10	0.38	± 0.048	~60	18	0.36	± 0.021
~70	21	0.58	± 0.071	~70	16	0.39	± 0.028	~70	22	0.37	± 0.024
~80	18	0.59	± 0.025	~80	18	0.36	± 0.035	~80	16	0.35	± 0.052
~90	9	0.57	± 0.031	~90	12	0.36	± 0.016	~90	14	0.6	± 0.037
100	6	0.87	± 0.022	100	6	0.91	± 0.011	100	6	0.85	± 0.049

**Table 5:** Abrasive wear factor test results.

1st Group of Samples				2nd Group of Samples				3rd Group of Samples			
*q* (%)	Number of Tests Performed	*K_c_* Value (10^−13^·m^3^·N^−1^·m^−1^)	Standard Deviation	*q* (%)	Number of Tests Performed	*K_c_* Value (10^−13^·m^3^·N^−1^·m^−1^)	Standard Deviation	*q* (%)	Number of Tests Performed	*K_c_* Value (10^−13^·m^3^·N^−1^·m^−1^)	Standard Deviation
0	12	4.264	± 0.724	0	12	4.574	± 0.548	0	12	5.134	± 0.417
~15	6	3.213	± 0.994	~20	8	3.341	± 0.221	~10	6	2.994	± 0.805
~20	18	3.115	± 0.347	~25	8	3.107	± 0.304	~25	14	2.743	± 0.572
~30	12	2.795	± 1.054	~35	6	2.824	± 0.047	~40	10	2.889	± 1.094
~40	15	2.845	± 0.943	~40	10	2.734	± 0.177	~45	8	2.671	± 0.902
~50	15	2.677	± 0.617	~50	12	2.841	± 0.097	~50	16	2.747	± 0.394
~60	18	2.549	± 0.448	~60	10	2.611	± 0.759	~60	18	2.703	± 0.615
~70	21	0.116	± 0.003	~70	16	2.594	± 0.607	~70	22	2.655	± 0.677
~80	18	0.121	± 0.047	~80	18	2.648	± 0.050	~80	16	2.611	± 0.284
~90	9	0.124	± 0.029	~90	12	0.106	± 0.036	~90	14	0.164	± 0.032
100	6	0.399	± 0.260	100	6	0.338	± 0.077	100	6	0.432	± 0.106

## Data Availability

The data presented in this study are available on request from the corresponding author. The data are not publicly available due to hardware limitations.

## References

[1] Elakhame Z.U., Olotu O.O., Abiodun Y.O., Akubueze E.U., Akinsanya O.O., Kaffo P.O., Oladele O.O. (2017). Production of Asbestos Free Brake Pad Using Periwinkle Shell as Filler Material. Int. J. Sci. Eng. Res..

[2] Matějka V., Metinöz I., Wahlström J., Alemani M., Perricone G. (2017). On the running-in of brake pads and discs for dyno bench tests. Tribol. Int..

[3] Plachá D., Vaculík M., Mikeska M., Dutko O., Peikertová P., Kukutschová J., Kutláková K.M., Růžičková J., Tomášek V., Filip P. (2017). Release of volatile organic compounds by oxidative wear of automotive friction materials. Wear.

[4] Belhocine A., Abu Bakar A.R., Bouchetara M. (2015). Thermal and structural analysis of disc brake assembly during single stop braking event. Aust. J. Mech. Eng..

[5] Dong H., Dai H., Geng Y., Fujita T., Liu Z., Xie Y., Wu R., Fujii M., Masui T., Tang L. (2017). Exploring impact of carbon tax on China’s CO 2 reductions and provincial disparities. Renew. Sustain. Energy Rev..

[6] Talati F., Jalalifar S. (2009). Analysis of heat conduction in a disk brake system. Heat. Mass. Transf..

[7] Kulikowski K., Szpica D. (2014). Determination of directional stiffnesses of vehicels’ tires under a static load operation. Eksploat. Niezawodn..

[8] Belhocine A., Bouchetara M. (2014). Structural and Thermal Analysis of Automotive Disc Brake Rotor. Arch. Mech. Eng..

[9] Borawska E., Borawski A. (2020). Influence of the Initial Speed of the Agricultural Tractor on the Brakes Heating Process During Emergency Braking. Heat Transf. Res..

[10] Manjunath T.V., Suresch P.M. (2013). Structural and thermal analysis of rotor disc of disc brake. Int. J. Innov. Res. Sci. Eng. Technol..

[11] Grkić A., Mikluc D., Muždeka S., Arsenić Ž., Duboka Č. (2015). A Model for the Estimation of Brake Interface Temperature. J. Mech. Eng..

[12] Eriksson M., Bergman F., Jacobson S. (1999). Surface characterisation of brake pads after running under silent and squealing conditions. Wear.

[13] Xian J., Xiaomei L. (2004). Friction and Wear Characteristics of Polymer-Matrix Friction Materials Reinforced by Brass Fibers. J. Mater. Eng. Perform..

[14] Borawski A. (2020). Conventional and unconventional materials used in the production of brake pads – review. Sci. Eng. Compos. Mater..

[15] Baltoin J.G., Neis P.D., Ferriera N.F. (2010). Analysis of the influence of temperature on the friction coefficient of friction materials. ABCM Symp. Ser. Mechatron..

[16] Chen L., Chen G.S., Chang J. (2015). An Insight to High Humidity-Caused Friction Modulation of Brake by Numerical Modeling of Dynamic Meniscus under Shearing. Lubricants.

[17] Scieszka S.F. (1998). Hamulce Cierne—Zagadnienia Konstrukcyjne, Materiałowe i Tribologiczne.

[18] Eriksson M., Bergman F., Jacobson S. (2002). On the nature of tribological contact in automotive brakes. Wear.

[19] Gawande S.H., Banait A.S., Balashowry K. (2020). Study on wear analysis of substitute automotive brake pad materials. Aust. J. Mech. Eng..

[20] Sinha A., Ischia G., Menapace C., Gialanella S. (2020). Experimental Characterization Protocols for Wear Products from Disc Brake Materials. Atmosphere.

[21] Amato F., Cassee F.R., Van Der Gon H.A.D., Gehrig R., Gustafsson M., Hafner W., Harrison R.M., Jozwicka M., Kelly F.J., Moreno T. (2014). Urban air quality: The challenge of traffic non-exhaust emissions. J. Hazard. Mater..

[22] Peikertová P., Filip P. (2015). Influence of the Automotive Brake Wear Debris on the Environment—A Review of Recent Research. SAE Int. J. Mater. Manuf..

[23] Samet J.M., Dominici F., Curriero F.C., Coursac I., Zeger S.L. (2000). Fine Particulate Air Pollution and Mortality in 20 U.S. Cities, 1987–1994. N. Engl. J. Med..

[24] Pope C.A., Burnett R.T., Thun M.J., Calle E.E., Krewski D., Ito K., Thurston G.D. (2002). Lung cancer, cardiopulmonary mortality, and long-term exposure to fine particulate air pollution. J. Am. Med. Assoc..

[25] Ramadan M.A., Khashaba M.I., Ali W.Y. (2011). Friction and wear of automotive friction materials. J. Egypt. Soc. Tribol..

[26] Stachowiak G.W., Batchelor A.W., Stachowiak G.B. (2004). Experimental Methods in Tribology.

[27] Genovese A., D’Angelo G.A., Sakhnevych A., Farroni F. (2020). Review on Friction and Wear Test Rigs: An Overview on the State of the Art in Tyre Tread Friction Evaluation. Lubricants.

[28] Wright N., Kukureka S. (2001). Wear testing and measurement techniques for polymer composite gears. Wear.

[29] Borawski A. (2016). Suggested Research Method for Testing Selected Tribological Properties of Friction Components in Vehicle Braking Systems. Acta Mech. Autom..

[30] Fildes J., Meyers S., Kilaparti R., Schlepp E. (2012). Improved ball crater micro-abrasion test based on a ball on three disk configuration. Wear.

[31] Shipway P., Hogg J. (2007). Wear of bulk ceramics in micro-scale abrasion—The role of abrasive shape and hardness and its relevance to testing of ceramic coatings. Wear.

[32] Szpica D. (2015). Simplified numerical simulation as the base for throttle flow characteristics designation. Mechanics.

[33] Mieczkowski G., Molski K. (2006). Stress field singularities for reinforcing fibre with a single lateral crack. Solid Mech. Appl..

[34] Varinauskas V., Diliunas S., Kubilius M., Kubilius R. (2013). Influence of cantilever length on stress distribution in fixation screws of all-on-4 full-arch bridge. Mechanics.

[35] Szpica D. (2016). Research on the influence of LPG/CNG injector outlet nozzle diameter on uneven fuel dosage. Transport.

[36] Byskov E. (1970). The calculation of stress intensity factors using the finite element method with cracked elements. Int. J. Fract..

[37] Borawski A., Szpica D., Mieczkowski G. (2020). Verification tests of frictional heat modelling results. Mechanics.

[38] Borawski A. (2020). Effects of initial speed and load of universal semitrailer on braking systems friction pair heating. Heat Transf. Res..

[39] Cozza R.C., Wilcken J.T.D.S.L., Schön C.G. (2018). Influence of abrasive wear modes on the coefficient of friction of thin films. Tecnol. Metal. Mater. Min..

[40] Gee M., Gant A., Hutchings I., Bethke R., Schiffmann K., Van Acker K., Poulat S., Gachon Y., Von Stebut J. (2003). Progress towards standardisation of ball cratering. Wear.

[41] Osuch-Słomka E., Słomka Z., Ruta R. (2013). The use of a modern method of designing experiments in ball-cratering abrasive wear testing. Proc. Inst. Mech. Eng. Part J J. Eng. Tribol..

[42] Shoemaker A.C., Kacker R.N. (1988). A methodology for planning experiments in robust product and process design. Qual. Reliab. Eng. Int..

[43] Lewandowski A., Using G. (2013). Taguchi’s Research Planning Method for Analysis and Optimisation of Cast Iron EN-GJLP-250 Surface Burnishing. Tribologia.

[44] Rao R.S., Kumar C.G., Prakasham R.S., Hobbs P.J. (2008). The Taguchi methodology as a statistical tool for biotechnological ap-plications: A critical appraisal. Biotechnol. J..

[45] Osuch-Słomka E. (2011). Proposed method for determining the values of tests for the ball-cratering metod. Tribologia.

[46] BSI (2007). BS EN 1071-6:2007 Advanced Technical Ceramics—Methods of Test for Ceramic Coatings—Part 6: Determination of the Abrasion Resistance of Coatings by a Micro-Abrasion Wear Test.

[47] Borawski A., Tarasiuk W. Comparative analysis of protective coatings of car paints. Proceedings of the IOP Conference Series: Materials Science and Engineering (KONMOT 2018).

